# A qualitative study of factors affecting mental health amongst low-income working mothers in Bangalore, India

**DOI:** 10.1186/1472-6874-14-22

**Published:** 2014-02-07

**Authors:** Sandra Mary Travasso, Divya Rajaraman, Sally Jody Heymann

**Affiliations:** 1 Division of Epidemiology, St. John’s Research Institute, St. John’s National Academy of Health Sciences, Bangalore 560034, India; 2 UCLA Fielding School of Public Health, Los Angeles, USA

**Keywords:** Low-income work, Mental health, Women’s health, India

## Abstract

**Background:**

Low-income urban working mothers face many challenges in their domestic, environmental, and working conditions that may affect their mental health. In India, a high prevalence of mental health disorders has been recorded in young women, but there has been little research to examine the factors that affect their mental health at home and work.

**Methods:**

Through a primarily qualitative approach, we studied the relationship between work, caring for family, spousal support, stress relief strategies and mental health amongst forty eight low-income working mothers residing in urban slums across Bangalore, India. Participants were construction workers, domestic workers, factory workers and fruit and vegetable street vendors. Qualitative data analysis themes included state of mental health, factors that affected mental health positively or negatively, manifestations and consequences of stress and depression, and stress mitigators.

**Results:**

Even in our small sample of women, we found evidence of extreme depression, including suicidal ideation and attempted suicide. Women who have an alcoholic and/or abusive husband, experience intimate partner violence, are raising children with special needs, and lack adequate support for child care appear to be more susceptible to severe and prolonged periods of depression and suicide attempts. Factors that pointed towards reduced anxiety and depression were social support from family, friends and colleagues and fulfilment from work.

**Conclusion:**

This qualitative study raises concerns that low-income working mothers in urban areas in India are at high risk for depression, and identifies common factors that create and mitigate stress in this population group. We discuss implications of the findings for supporting the mental health of urban working women in the Indian context. The development of the national mental health policy in India and its subsequent implementation should draw on existing research documenting factors associated with negative mental health amongst specific population groups in order to ensure greater impact.

## Background

Mental health disorders contribute significantly to the global burden of non-communicable chronic disease: the World Health Organization has estimated that mental and behavioural disorders account for 12% of the global burden of disease [1], while community-based epidemiological studies in different settings have recorded a lifetime prevalence of mental disorders ranging from 12.2–48.6%, with a 12-month prevalence from 8.4-29.1% [2]. Common mental disorders can increase risk for communicable diseases (HIV/AIDS, tuberculosis), non-communicable diseases (cardio vascular diseases, diabetes), and injuries, and negatively impact maternal and child health [3]. While increasing attention is being directed towards reducing the burden of non-communicable illnesses such as cardio-vascular disease, cancer, diabetes and chronic respiratory disorders in low- and middle-income countries [4], much less has been done to provide mental health services to tackle common mental disorders [5,6].

Low-income urban working mothers face a range of challenges in their domestic, environmental, and working conditions that may affect their mental health. In India, the second most populous country in the world, approximately 31% of the total population lives in urban areas [7] and almost a quarter of urban residents live in slums [8]. To meet financial needs, the majority of women work, but they commonly have limited job choices and frequently cite heavy workload challenges [9]. Research suggests that life stressors associated with poverty increase the risk of mental health disorders [10-12], and that women are more vulnerable than men to mental health problems such as unipolar depressive disorders, schizophrenia, unipolar affective disorders and self-inflicted injuries [1]. While urbanization brings many social and economic opportunities for women, men, and families, the urban poor face a particularly challenging socio-physical environment, which can take a toll on their mental health [11,13]. The suicide rate in India is among the highest in the world, at 3% of all deaths, 56% of which are amongst women [14]. One of the largest population-based studies of mental health in India was carried out in urban South India among 26,001 participants, and recorded a 15.1% prevalence of depression in the general population. Depression was reported at higher rates amongst women (females 16.3% vs. males 13.9%) and low-income individuals (low-income group 19.3% vs. high-income group 5.9%) [15].

A few small-scale initiatives to provide community-based mental health services in India have been successful [16,17], but the need for well-designed and appropriately targeted services for a range of different population groups remains great. Filling this need will require evidence on both the triggers of poor mental health and the potential mitigators. Various studies in urban India have identified risk factors for depression and suicide among people living in slums: an ethnographic study set in Mumbai slums conducted amongst a small sample of men and women found that poverty, poor living and working conditions, alcoholic husbands and intimate partner violence were factors that increased risk for depression [11], while another rural study identified husband’s alcohol consumption, intimate partner violence and financial difficulties as increasing risk for attempted suicide among women [18]. A socio-cultural autopsy conducted in urban slums in Mumbai among family members of people who committed suicide identified financial stressors, marital problems, and alcohol use as common themes in suicidees’ lives [19]. Another study conducted in urban slums in India revealed that 63.31% of the 443 participants suffered from serious mental health issues which were related to financial problems, marital conflicts, interpersonal conflicts and housing problems [20].

While a number of studies have considered factors associated with mental health in rural and urban settings in India, this is the first qualitative study that analyses the specific needs of low-income working mothers in urban areas in India. In this paper, we examine life stressors as well as stress mitigating factors amongst low-income working mothers in Bangalore, the fifth most populous city in India [7], in order to understand factors affecting mental health in this growing population, and inform strategies to address them.

## Methods

### Study design

Data for this analysis is drawn from a project aimed at understanding how low-income urban working mothers balance child care and work obligations within the context of family and community support, working conditions, children’s health and education, and their own health needs. Therefore, women of reproductive age were recruited for the study. This paper reports specifically on how the interplay of these factors affects mental health, assessed both qualitatively through descriptions of well-being and sadness, as well as quantitatively through the Kessler-10 scale.

### Study setting and participants

Mothers of children between the ages of 0-8 years old who were currently working (and had been working for a minimum of a year) as construction workers, domestic workers, garment factory workers, or fruit/vegetable/flower street vendors were eligible to participate in the study. The four occupation groups were selected on the basis of being the most common low-income occupations amongst mothers of young children living in slums in Bangalore, and were initially identified through discussions with key informants (women’s group leaders in slums; representatives of NGOs working on labour issues, community development, women’s rights and child rights; government day care centre workers in slums; and, representatives of trade unions for low income workers). These occupations also represented a range of working conditions, such as variable work hours and multiple employers for domestic workers; daily wage labour for construction workers and some street vendors; self-employment and flexible working hours for some street vendors; and, unionization and industrial labour regulation for garment factory workers. The appropriateness of the selected occupations was validated through rapid mapping of women’s occupations and incomes on visits to 5 small/medium slums in the city. Other common women’s occupation groups that were encountered during these visits were street sweepers and incense makers. Street sweepers were not included as part of the study because the majority of street sweepers were older and did not have children between 0-8 years. Incense rollers were not included as part of the study because they worked short and flexible hours at home, and consequently did not face the same challenges balancing work and home responsibilities as other working mothers. Amongst the selected occupation groups, monthly income for a full day’s work ranged from INR4,000-INR8,000 (USD75-150).

A qualitative researcher visited multiple worksites (2 construction sites, 4 fruit and vegetable markets, 3 garment factories) and low-income residential areas (22 medium to large slums across Bangalore) to recruit study participants who would represent a range of working and living conditions. Forty-eight women (12 from each occupation group) who met the inclusion criteria were recruited.

### Ethics statement

The study protocol received ethical approval from the institutional review board of the St. John’s Medical College, Bangalore, India. The purpose and procedures of the study were verbally explained to participants, and they were advised that they could choose not to answer any of the questions or withdraw from the interview at any time. Information sheets were available to literate participants, and all participants signed or initialled an informed consent form. A small cash honorarium of a hundred Indian rupees (USD1.60) was provided to participants, as compensation for their time.

### Data collection and analysis

Interviews were conducted by a qualitative researcher in the local language, Kannada, at a time and place of the participant’s choice. To maintain confidentiality, a private location was sought for the interview; however, some participants selected locations where they could continue to do their work, even if others could hear the conversation. The data was collected in 2 phases between August-November 2011 (24) and December 2011-January 2012 (24). All participants (n = 48) were administered a short closed-ended questionnaire. The closed-ended questionnaire was developed for this study to include basic indicators to assess the demographic and economic profile of the household. It included questions on participant age, marital status, participant and spouse highest education level, years of experience in current occupation, house ownership, number and age of children, monthly household income, household assets (mobile phone, bicycle, motorbike, car/auto rickshaw, television, refrigerator), and workplace benefits (Provident Fund, paid leave, maternity leave, health insurance). The participants were then interviewed with in-depth open ended questions for about an hour about their working conditions, child care and spousal support, child and maternal health and wellbeing, and family, community and other types of support available to them. Following a preliminary analysis of the first phase of interviews, more detailed questions were added in the second phase on mental health status, the relationships between work, caring for family, spousal support and mental health, the availability of emotional support, and stress mitigators. The Kessler psychological distress questionnaire was also administered to the second phase participants. This 10-item scale, validated for use in India, yields a global measure of distress based on questions about anxiety and depressive symptoms that a person has experienced in the past four weeks [21,22]. Participants who were experiencing substantial mental distress were given the name, location and contact details of local NGOs that provided counselling and other support services for women.

After completion of data collection, all participants were assigned aliases to maintain anonymity in the reporting of results. Interviews were simultaneously translated and transcribed in English by bi-lingual research assistants. The transcripts were reviewed by the interviewer, who listened to the audio recording while reading through the transcripts to check for accurate and consistent translation. Transcripts were subsequently language edited to ensure contextual translation. The transcripts were then analysed thematically, using NVivo 9.2 qualitative data analysis software [23]. Transcripts were coded for descriptions of state of mental health, factors that affected mental health positively or negatively, manifestations and consequences of stress and depression, and stress mitigators. Mental health concerns were qualitatively identified when respondents spoke about life events or their general state of being in terms of feelings of severe stress, anxiety, sadness and despair, uncontrollable crying, persistent frustration, feelings of worthlessness, no desire to live and suicide attempts.

The socio-economic and Kessler scale data were entered and analysed in Statistical Package for the Social Sciences v.18 [24]. A variable was created to assess if the participants were below poverty line, based on a new methodology proposed by the Planning Commission, which takes into account residential, occupational and social vulnerability of families [25]. Although we did not have all the information necessary to create a vulnerability score for participants, we were able to categorize families living below the poverty line based on automatic inclusion criteria related to occupation of participants and their spouses.

## Results

### Socio demographic and economic characteristics

Study participants were aged between 19 to 40 years, and had between 1 and 5 children. Socio-demographic and economic characteristic of the participants are detailed in Table 1. The participants were characterized by low education levels. Out of 48 participants, 40 were married, 5 were widowed and 3 were divorced or separated. The monthly household income ranged from INR 2,000-12,000 (USD 37-225). Based on the automatic inclusion criteria for identifying poor urban households [25], 42 of the 48 respondents were categorized as living below the poverty line. These included households where any member is a domestic worker (12), where all earning adults are daily wagers or irregular wagers (24), women headed households (2), and households where the main bread-winner is a head-loader, security guard, driver or painter (4). Most women had been working in their current occupations for several years (median 8 years, range 1-22 years).

**Table 1:** Socio-demographic and economic characteristics of the participants

	**Construction worker *(12)***	**Domestic worker *(12)***	**Factory worker *(12)***	**Street vendor *(12)***	**Total *n = 48***
**Age in years (median, range)**	25, 20-35	28, 21-36	25, 21-31	21, 19-40	25, 19-40
**Marital status**					
Married	11	11	10	8	40
Widowed	-	1	2	2	5
Divorced/separated	1	-	-	2	3
**Number of children (median, range)**	3, 2-5	3, 1-5	2, 1-4	2, 1-5	3, 1-5
**Respondent higher education level**					
No education	7	4	3	5	19
Primary	4	7	6	7	24
Secondary	1	1	2	0	4
Tertiary	0	0	1	0	1
**Spouse highest education level**					
No education	6	2	2	2	12
Primary	3	7	5	4	19
Secondary	2	2	1	2	7
Tertiary	1	0	2	0	3
**Number of years in current occupation (median, range)**	12, 2-20	8, 1-22	6, 2-14	8, 3-20	8, 1-22
**Monthly household income in INR (range)**	2,000 -10,000	2,800 – 11,000	2,000 – 12,000	3,000 – 10,000	2,000 – 12,000
**Below poverty line**	12	12	6	12	42

### Mental health status

Out of the 24 respondents who were administered the Kessler 10 test, nearly half (11) were diagnosed as likely to have a moderate to severe mental disorder. One third (8) were likely to have a mild mental disorder, and only a fifth (5) were diagnosed as likely to be well. In the qualitative interviews with all 48 participants, about two thirds of women (32) mentioned that their mental health was not good. Less than one fifth of the participants (9) mentioned that they felt peaceful or happy with the current situation in their life. Amongst Phase 2 participants, 17 of the 21 women who spoke about their mental health concerns in interviews were found likely to have a mild to severe mental disorder as per the Kessler scale. This indicates a correspondence of over 80% between qualitative assessment and the Kessler assessment.

Some women had experienced sustained states of depression and hopelessness, while others spoke about experiencing a poor state of mental health which had passed when circumstances changed. The mothers in this study described a range of symptoms commonly associated with manifestations of poor mental health (detailed in Table 2). At the extreme end were attempts at suicide (2), suicidal ideation (5) and uncontrollable crying (14). A large number of respondents also mentioned feelings of anxiety, stress, frustration, disappointment, despair and reduced desire to live. Consequences of this stress reported by respondents included inability to care properly for family (21), hitting children (18), poor performance at work or missing work altogether (8), picking fights with spouse and family members (6), lack of desire to interact with others (4) and chewing tobacco (2). None of the participants mentioned seeking professional help for anxiety or depression. The major causes of poor mental health and stress mitigating factors identified by respondents are discussed below.

**Table 2:** Symptoms and consequences of poor mental health amongst study participants, as mentioned during in-depth interviews

	**n = 48**	**%**
**Symptoms of poor mental health**		
Uncontrollable crying	14	29.2
Suicidal ideation	5	10.4
Attempted suicide	2	4.2
**Consequences of poor mental health**		
Inability to care properly for family	21	43.8
Hitting children	18	37.5
Poor performance at work or missing work altogether	8	16.7
Picking fights with spouse and family member	6	12.5
Lack of desire to interact with others	4	8.3
Chewing tobacco	2	4.2

### Factors contributing to mental distress

The most frequently cited factors that affected mental health negatively were an alcoholic spouse, intimate partner violence, poor working conditions, and barriers to caring for children. While most women in the study reported experiencing these factors at one time or another, long-term prevalence of mental distress appeared to be greatest amongst those facing sustained difficult circumstances such as having an alcoholic spouse, on-going intimate partner violence, the need to care for special needs children, or lack of support for providing quality routine care for children. Women who experienced passing mental health disorders were often affected by health and financial shocks caused by severe illness in children, or by a combination of stressors occurring simultaneously. Women who experienced the resolution of some or all of the stressors often perceived improvements in their mental health state.

### Alcoholic spouses and intimate partner violence

More than half the women (25) in the study reported that their husbands drank alcohol regularly and heavily. Most of the women whose husbands drank regularly also reported experiencing intimate partner violence (22), which profoundly affected their mental health. Most of the respondents (5 of 7) who spoke about attempted suicide or suicidal ideation were victims of intimate partner violence. In some families, children were also victims of abuse by an alcoholic father. None of the women mentioned seeking help in dealing with an alcoholic spouse or intimate partner violence.

Bhavya related how constant verbal and physical abuse from her alcoholic husband had pushed her to attempt suicide, and continued to be a source of domestic strife:

“My daughter was very small at that time. One night [my husband] came and beat me a lot and told me to leave the house. I went to the railway station and tried to commit suicide on the railway track. One of my relatives saw me there and took me back [home], telling me: ‘a drunkard’s words are not serious, in the morning it will be alright.’ Another time I took off my mangalsuthra (necklace symbolising marriage in Hinduism) and gave it to him, saying ‘I don’t want you,’ and went to my sister’s place. I stayed there for one week, he came to me after a week and asked for forgiveness and took me back [home]. Even last night he was drunk and fought with me till midnight. When he comes home drunk, he tells me to leave the house, sometimes he asks me to go out alone, sometimes he asks me to take my children also”. Bhavya, Construction Worker

Manjulla spoke about wishing she could die when her husband abused her, but recognized the need to live to take care of her children:

“I feel ‘why should I be abused by him, let me die,’ but seeing my children’s faces, I think: ‘I am alive, if I die my mother-in-law might take care for some days, [what] after that? My husband will get married again, he will not take care of my children.’’ Manjulla, Domestic Worker

Sruti, a 19 year old mother of a six month old infant, has suffered significant mental distress as a result of spousal abuse, but had experienced relief upon separating from her husband:

*“I am at peace now, that’s what I feel because when my husband was around he used to fight everyday and I had no peace of mind. But for the past one month (*after separating*), now I am at peace”. Sruti, Street Vendor*

Mahima, a 28 year old mother of 3 children spoke about the physical and verbal abuse that her children endured due to their father’s alcoholism. To protect them, she sent her children away to boarding school. She worries that her children are not receiving adequate care and nutrition at school, but feels that she cannot keep them at home because of her abusive spouse:

“My husband is an alcoholic, when he drinks and comes home he uses bad words in front of the children and that has an effect on the children…. Besides that, my husband hits the children a lot or sometimes when he hits me my children start crying and they come to stop the father, but my husband does not bother and he hits the children also. I feel bad to see them getting beatings…so I sent them away to a hostel”. Mahima, Domestic Worker

#### Challenges providing for children’s health needs

Mothers with special needs children are particularly susceptible to chronic stress and depression. Most respondents also reported that they faced barriers in providing adequate care when their children fall sick, including not having enough money for treatment and not having time to care for the sick child due to work commitments. These ill-health episodes could cause significant mental anguish, leading to short-term anxiety and even depression. When Lalitha, a mother of two children, had to care for her 4 year old daughter who fell sick, she had to take out loans to pay for her daughter’s health care, and did not have money to pay for transportation to bring her daughter home from the hospital, despite working hard to earn an income to support her family; this contributed to her feelings of hopelessness:

“When my elder daughter was not feeling well, I didn’t eat for 2-3 days, I was crying the whole day and sitting on the road, while she had to come back from hospital at 3 pm. Somebody helped me by giving me a ride in their vehicle at night…..I didn’t have money, I had pledged my mother’s jewellery and taken her to the hospital, I felt very bad that time, I didn’t have money and my daughter was very serious, at that time, I felt, why should we be living?” Lalitha, Domestic Worker

Financial difficulties, lack of support, and devaluation of the lives of children with special needs affected both the mothers and their children. Sameena, a 31 year old mother of four girls, found it difficult to cope with caring for her 8 year old daughter who was born with a heart defect, given her financial constraints and the lack of support from her alcoholic husband. She took six months off from work to get the child treated, which worsened her economic situation, and increased her feelings of frustration and extreme despair:

“The third child had a heart problem. She was very weak, that’s why she had a problem in her growth. I really had a tough time in caring for that child. I really suffered a lot with my alcoholic husband. And, seeing my financial problems, many [people] used to suggest that I kill this baby”. (Crying). Sameena, Factory worker

Roona, mother of two daughters and two mentally retarded sons, spoke about how she struggled with the responsibilities of caring for two special needs children, given her tenuous financial situation and her unhelpful and sometimes abusive husband:

“I feel bad about my condition. I have a lot of financial problems, health problems. God has given me two mentally retarded children. At least I should have had one normal boy child; that really makes me feel low, especially when I fall sick and my husband gives no support in looking after children. Instead of cleaning (the mess the children make), he calls me. So often, I feel that I have problems with my children and even with my husband. So I get angry with the children and hit them. Today morning, too, I cried thinking of my situation”.

Roona shared these circumstances that she felt had made her chronically depressed and described her two suicide attempts: she was rescued once by her neighbours and once by her 12 year old daughter.

“I was really depressed. It was 4 years ago. I was 3 months pregnant and I tried to commit suicide. I had closed the door so that no one would be able to see me hanging. Usually I don’t close the main door in the evening so my neighbours got suspicious and they came in looking of me and they broke the door. I was hanging and struggling so they came and rescued me for the second time, and my husband ran away from the city and came back after 4 months”. Roona, Domestic Worker

#### Challenges providing for routine child care

Amongst working mothers, routine child care concerns could also be a cause of anxiety and stress. Respondents expressed a number of concerns about their children’s well-being, including abusive childcare, children’s safety in the absence of supervised child care, financial and time constraints to providing adequate nutrition and supervision for children, and lack of physical and emotional support to care for their children.

Anjali, a factory worker who had placed an infant daughter in her workplace day care centre had worried daily that the day care providers were giving the child medications to make her sleep. Similarly, Radhika, a domestic worker and mother of two girls and two boys, who had sent her pre-school age children to a free government child care centre, was concerned about abusive care providers:

“If we leave them with someone else, they hit and scold when they feed the children. So when I see all that, I don’t feel like leaving them there. Only a few people might take care well, not everyone. I feel bad…(about it)…being a mother”. Radhika, Domestic Worker

Aandal, a construction worker with an 11 year old son and a 6 year old daughter, leaves her children at home while she is at work because of lack of alternative childcare, and experiences constant fear about her younger child’s safety:

“I worry a lot, I’m always thinking about what she (my daughter) is doing, vehicles are moving on the street…whether she is safe, I have fear”. Aandal, Construction Worker

Eeswari, a 25 year old mother of 5 children, was forced to carry her children to her workplace and feels extremely physically and mentally stressed due to the strenuous work at construction sites and unsympathetic contractors who wouldn’t let her care for her children at the work site. She has to work even when she is injured at work or sick so that she doesn’t miss her daily wage. She spoke about hitting her children when she is stressed; she also admits eating *“pan”* betel leaf to cope with stress:

“Sometimes when I get really irritated I just hit the children and they cry for some time. Most of the time if I get tense, I take it out on my children”. Eeswari, Construction Worker

Rajeswari a 23 year old mother expressed her frustration about the lack of financial, emotional and physical support for raising her two daughters:

“Sometimes I sit alone and cry. I don’t want children or my husband…no one, I feel like running away somewhere. My husband is a big problem, if he works properly I will not have any problem. From where shall I bring money, children are growing, thinking all this I am not able to eat properly. Both are daughters. How to bring them up, I don’t have support from any one, so I worry a lot. Nobody comes and supports us. So I feel very disappointed about it”. Rajeswari, Construction Worker

Meena, a 36 year old mother of 5 children, was one of several respondents who expressed helplessness and distress due to not being able to provide her children with appropriate nutrition:

“I feel shame…because I go to work and wouldn’t know how [my children] eat, there is no time, every mother ensures that their children are eating at school, but we do not have that fortune, we go to work, we have left it up to them, but I feel, that they don’t eat properly, they eat as they want…I have just left it upon God’s mercy, what to do we have difficulty. We have to work to take care of them”. Meena, Domestic Worker

#### Work conditions

The women in our study expressed how negative working conditions also affect their mental health. Domestic workers had no formal leave from work. This was a source of anxiety when women had to take long leaves because of their own or children’s health problems, and they worried about losing their job as a consequence.

Selvi was denied leave when she was having a high fever and feeling too weak to stand, she narrated an instance when she had an injury at work and her employer refused to give her leave:

“I had gone to the doctor and he gave me an injection. The next day I didn’t go to work. “For this small wound you didn’t come to work? I thought you were not feeling well. If your son is not feeling well we need to give you leave, if you daughter is not feeling well we need to give leave?” They (employers) scold”. Selvi, Domestic Worker

Some factory workers spoke about how they were affected by the reprimands of their supervisors if they did not achieve production targets:

“They (supervisors) have scolded us. They have scolded us many times. I do make mistakes, we might have missed something and sent [the garment]. Because of that they scold us many times. We have felt bad many times, we have cried, sometimes at night we don’t get sleep. We feel so bad”. Bhanu, Factory Worker

Construction workers reported instances of abuse from drunken male co-workers and contractors, and contractors threatening them if they lodged any complaints about the physical strain of the work.

“If I complain anything about work [the contractors] beat us..... They don’t even feel bad (about beating us)…. So we don’t tell anyone that they beat us like that.…If they get to know that I am speaking against them they will kill me. That’s why I just go, do my work and come back. We borrow money from them, so they call us whenever there is work and they take money from us. Many times we have health problems, so we take a loan”. Aleyamma, Construction Worker

“If we tell [the contractor] ‘I can’t do this work,’ [the contractors] say ‘If you cannot work, why have you come here, stay at home.’ If I am at home, how can we live our life? We have to do the work that they tell us to do”. Nisha, Construction Worker

### Mitigators of stress

#### Supportive spouse

Having a supportive spouse who contributes to household expenses, participates in childcare and helps in household tasks was a significant mitigator of life stressors. A few respondents (8) mentioned that their husband helped them with household chores when they were unwell. Many respondents (31) also mentioned that their husband contributed money to meet the household expenses, although in most cases, insufficiently or irregularly. Some of the respondents (12) mentioned that that their husband helped to take care of the children on a routine basis, which reduced their concerns about child care. This was described by a street vendor:

“In the afternoon my husband comes for lunch so he takes [our daughter] out and spends time with her so if my husband is there I need not worry at all about her”. Alaka, Street Vendor

#### Supportive work environment

While a few women spoke about work as being a stressor, empathetic employers and friendly colleagues were commonly mentioned as factors that helped to relieve stress caused by problems at home. More than a quarter of the respondents (14) mentioned that work had a positive impact on their mental health. These women discussed problems related to children, spouse, work or their general state of mind with their employers or colleagues, and found the sharing to be very effective in relieving stress and alleviating depression. The respondents who reported that work had a positive impact on their mental health all spoke about experiences of sharing their feelings, especially when they are facing difficult times at home. A few examples are cited below:

“Just this morning, I cried thinking about my situation. For the coming festival, all children will wear good clothes but I have no money to spend for my children. I can’t give them clothes. I felt I should die and I should not live when I can’t fulfil my children’s desires. When I went for work, my employer felt bad about my situation and she gave me 700 Rupees to get new clothes for my children. She listens to my problem and acts accordingly, so I feel a little relaxed. She is more than a mother to me, whenever I am very depressed I don’t even share with my mother but I share with my employer”. Mahima, Domestic Worker

“If I come to work, everyone here talks nicely to me and I can share my feelings with them and people crack jokes and spend time in a very jolly manner so I get a feeling that if I come to work I can forget a few bad things which have happened in my life and be happy for sometime at least. If I am alone I start thinking about so many things and I get disturbed mentally, so I feel that it is good for me if I can come to work since I forget the tensions I have and involve completely with work that gives me peace of mind”. Devi, Street Vendor

“Only if I go to work I feel peaceful, if I don’t go to work I don’t feel peaceful”. Rajeshwari, Construction Worker

“If I have too many problems and I am in tension, [my colleagues] ask me ‘What is wrong with you, why you are sitting like this? Only if you share, you will feel better.’ They try to console me”. Khadija, Factory Worker

“I share with my colleagues what is happening in my family and they tell me not to worry about such things in life and console me. I keep saying that I want to die and all, so they tell me ‘you have two children, so you should not think like that.” Anjali, Factory Worker

## Discussion

This qualitative study raises concerns that low-income working mothers in urban areas in India are at a high risk for depression. Women who have an alcoholic and/or abusive husband, face intimate partner violence or are raising children with special needs appear to be more susceptible to severe and prolonged periods of depression and suicide attempts. At the same time, when a range of more temporal life stressors (such as illness in the family especially among children, financial problems, and lack of support from family members) strike at the same time, this could push women into spells of severe depression. Even within our small study sample, extreme consequences of depression were evident: several women spoke of instances of attempted suicide and suicidal ideation. None of the women in the study, including those who attempted suicide, mentioned seeking professional support for anxiety or depression. There were a number of mitigating factors that could help women to battle anxiety and depression, such as social and financial support from family, friends and colleagues and the distraction or fulfilment of work. Given the need to address mental health issues amongst women in India, these findings suggest potential directions for mental health interventions among low-income working mothers in urban India.

Firstly, intimate partner violence, usually associated with spouses’ alcohol consumption, was reported as one of the most frequent causes of poor mental health by this study population. The relationship between intimate partner violence and depression is widely documented at a global level. Most of the women in our study who spoke about suicidal ideation or suicidal attempts have been victims of intimate partner violence. A WHO multi-country study in other low- and middle-income countries (study did not include India) showed that intimate partner violence has a strong association with suicidal attempts in women [26]. Another systematic review of studies conducted globally, including India found that intimate partner violence was associated with incident depressive symptoms and incident suicide attempts [27]. Intimate partner violence has been widely documented in India [28-30], and the levels are extremely high: in a nationally representative survey, 40% of reproductive age Indian women reported having experienced intimate partner violence, with the prevalence being much higher in lower socio-economic groups [31]. While a large number of NGOs in India have responded to the high prevalence of intimate violence with counselling services and shelters for abused women, awareness of these services remains low, and access uneven. Furthermore, providing services to victims of domestic violence is not a sufficient strategy in and of itself, without also addressing community and structural drivers such as women’s power, male identity and community norms, including family elders’ roles in condoning or instigating domestic violence [32,33]. In our study, none of the participants mentioned seeking help to stop intimate partner violence. There remains an urgent need in India to systematise and scale up screening for intimate partner violence in health care facilities and other institutions such as workplaces, and to introduce firm linkages between identification of intimate partner violence and mental health support services [18]. A promising intervention that has been piloted in southern India is the Dil Mil intervention study, which identifies participants through primary health clinics and aims to protect young women who are experiencing domestic violence by delivering intergenerational counselling sessions to mothers-in-law and daughters-in-law [34]. In addition to mental health and intimate partner violence services, economic empowerment services may have a role to play. The IMAGE trial in South Africa indicated that combining microfinance based poverty alleviation program and participatory training on gender and intimate partner violence can lead to economic and social empowerment and reduced violence from intimate partners [35]. It is unknown whether this will work in India but some research suggests that economic empowerment coupled with higher education levels is protective against partner violence [36].

Secondly, while routine child care concerns were a source of anxiety for mothers of healthy young children, the emotional and physical demands of caring for special needs children appeared to be associated with long-term depression and worse mental health, especially amongst mothers who received little help from their spouses. This finding is consistent with research conducted amongst low-income parents of special needs children in the United States. In industrialized countries, employer education and labour legislation have been recommended as strategies to address these issues [37]. However, given the large proportion of women who work in the informal sector in India, an avenue that may yield greater benefits is focussing on schools for special needs children as a location for introducing and advertising ancillary support for caregivers such as counselling services, telephone help lines, and support groups for parents. Also, low cost support for parents in community settings provided by non-specialist trained community workers has shown promise as a first line of support within comprehensive “packages of care” [18,38]. Mental health support in the community should include sharing experiences that could help to reduce parents’ feelings of social isolation [39-41], with strong linkages and referral systems to mental health professionals who could provide access to services such as cognitive behavioural therapy as well as treatment for depression where required [42,43].

Thirdly, with declining access to extended family support, working mothers of pre-school aged children face challenges in accessing adequate child care, particularly when spouses were unsupportive. Concerns about child abuse, nutrition and safety were a source of significant anxiety for these mothers. Government policies and strategies to improve the quantity and quality of safe and affordable childcare could play a large role in supporting better health outcomes for mothers and children alike.

Lastly, our study examined the role of supportive work environment particularly in relation to psychological benefits of emotional support offered by colleagues. In India, data on the effects of work on the mental health of mothers is virtually non-existent. In our study, many of the mothers who had school-aged children spoke about the psychological benefits of their work: these included support when their employers were sympathetic and helpful, the chance not be exposed to and focus on the problems at home for some hours of the day, and, most of all, the emotional support and encouragement they received from their colleagues when they spoke about their problems, including depression and suicidal ideation. These findings suggest that, apart from economic benefits, working outside the home can promote the mental health of mothers with children who are school-age, and pre-school age when care is available.

A strength of our study is that we examined women’s perspectives on factors that have impacted their mental health across their family and work roles. Previous research in India has not detailed the role of work in women’s mental health and how this interplays with women’s family responsibilities. Our study thus provides new and potentially more holistic insights into a combination of life stressors and triggers that can challenge the psycho-social resilience of low-income working mothers in urban India, as well as coping strategies to alleviate these. Our study findings should be interpreted in light of the following limitations: the study sample was small, limited to one city, and focused on four occupations groups, which limits the generalizability of the findings. Furthermore, the cross-sectional nature of data collected limits causal inference about the factors that triggered depression in this population. Keeping these limitations in mind, the findings nevertheless suggest important areas for further investigation through quantitative surveys and longitudinal intervention studies, while also highlighting areas for programme and policy action.

## Conclusion

India was one of the first of the low-income countries to recognize the need to address mental health with its National Mental Health Programme (NMHP) which was launched in 1982. Unfortunately, its goals remain largely unattained [44]. One of the reasons for this is the poor linkages between different levels of care and the weak relationship between health care facilities and the communities they serve [45]. India has now resolved to frame a mental health policy that takes into account the specific local context of mental illness [46]. The development of this policy and subsequent implementation should draw on existing research documenting factors associated with negative mental health amongst specific population groups, in order to ensure greater effectiveness and impact. Indeed, the Millennium Development Goals to improve maternal health, reduce child mortality, promote gender equality and empower women and eradicate extreme poverty cannot be attained without a specific focus on women’s mental health [47].

## Competing interests

The authors declare that they have no competing interests.

## Authors’ contributions

ST conducted the data analysis, wrote the first draft, and finalized the manuscript. DR conceptualized the study, supervised the data collection, provided guidance on data analysis, and co-wrote the final manuscript. JH conceptualized the study, provided guidance on data collection and analysis, and co-wrote the final manuscript. All authors read and approved the final manuscript.

## Pre-publication history

The pre-publication history for this paper can be accessed here:

http://www.biomedcentral.com/1472-6874/14/22/prepub
